# Deep learning for automated boundary detection and segmentation in organ donation photography

**DOI:** 10.1515/iss-2024-0022

**Published:** 2024-08-20

**Authors:** Georgios Kourounis, Ali Ahmed Elmahmudi, Brian Thomson, Robin Nandi, Samuel J. Tingle, Emily K. Glover, Emily Thompson, Balaji Mahendran, Chloe Connelly, Beth Gibson, Lucy Bates, Neil S. Sheerin, James Hunter, Hassan Ugail, Colin Wilson

**Affiliations:** NIHR Blood and Transplant Research Unit, Newcastle University and Cambridge University, Newcastle upon Tyne, UK; Institute of Transplantation, The Freeman Hospital, Newcastle upon Tyne, UK; Centre for Visual Computing and Intelligent Systems, Faculty of Engineering and Informatics, Bradford University, Bradford, UK; Department of Research Software Engineering, Newcastle University, Newcastle upon Tyne, UK; Translational and Clinical Research Institute, Newcastle University, Newcastle upon Tyne, UK; Nuffield Department of Surgical Sciences, University of Oxford, Oxford, UK

**Keywords:** transplant surgery, image segmentation, computer vision, deep learning, artificial intelligence

## Abstract

**Objectives:**

Medical photography is ubiquitous and plays an increasingly important role in the fields of medicine and surgery. Any assessment of these photographs by computer vision algorithms requires first that the area of interest can accurately be delineated from the background. We aimed to develop deep learning segmentation models for kidney and liver organ donation photographs where accurate automated segmentation has not yet been described.

**Methods:**

Two novel deep learning models (Detectron2 and YoloV8) were developed using transfer learning and compared against existing tools for background removal (macBGRemoval, remBGisnet, remBGu2net). Anonymised photograph datasets comprised training/internal validation sets (821 kidney and 400 liver images) and external validation sets (203 kidney and 208 liver images). Each image had two segmentation labels: whole organ and clear view (parenchyma only). Intersection over Union (IoU) was the primary outcome, as the recommended metric for assessing segmentation performance.

**Results:**

In whole kidney segmentation, Detectron2 and YoloV8 outperformed other models with internal validation IoU of 0.93 and 0.94, and external validation IoU of 0.92 and 0.94, respectively. Other methods – macBGRemoval, remBGisnet and remBGu2net – scored lower, with highest internal validation IoU at 0.54 and external validation at 0.59. Similar results were observed in liver segmentation, where Detectron2 and YoloV8 both showed internal validation IoU of 0.97 and external validation of 0.92 and 0.91, respectively. The other models showed a maximum internal validation and external validation IoU of 0.89 and 0.59 respectively. All image segmentation tasks with Detectron2 and YoloV8 completed within 0.13–1.5 s per image.

**Conclusions:**

Accurate, rapid and automated image segmentation in the context of surgical photography is possible with open-source deep-learning software. These outperform existing methods and could impact the field of surgery, enabling similar advancements seen in other areas of medical computer vision.

## Introduction

Medical image segmentation is a pivotal process in the field of medical imaging, serving as a fundamental technique for analysing and interpreting images obtained from various medical imaging systems such as Magnetic Resonance Imaging (MRI), Computed Tomography (CT), ultrasound and photographs from cameras. It involves partitioning an image into multiple meaningful regions or segments to extract regions of interest. This segmentation process plays a crucial role in medical diagnosis, treatment planning, image-guided interventions and disease monitoring. The potential options for image segmentation encompass a broad spectrum of methodologies ranging from simple thresholding and contour-based approaches to more sophisticated deep learning algorithms.

Over the years, medical image segmentation has witnessed significant advancements [1], driven by progress in imaging technology, computational methods and artificial intelligence (AI) techniques. Traditional segmentation approaches [2] often relied on manual or semi-automatic methods, requiring extensive user input and expertise. However, with the emergence of AI-driven techniques, particularly deep learning–based approaches, there has been a paradigm shift towards more automated and accurate segmentation methods. Convolutional neural networks (CNNs) and their variants, such as U-Net [3] and Mask R-CNN [4], have demonstrated remarkable performance in medical image segmentation tasks, achieving human-level or even superhuman-level accuracy in certain applications [5], 6].

Medical photography at time of retrieval has become an important adjunct for decision making in organ transplantation. Images of donated organs are routinely captured for documentation purposes and to facilitate remote quality assessment by implanting surgeons [7], [8], [9], [10], especially when retrieval is performed by a different team to that which will be implanting the donated organ. Organ utilisation decisions are nuanced, complex and multifactorial, with both clinical and non-clinical factors explaining why a retrieved organ may not be transplanted. They include reasons such as aberrant anatomy, organ damage, organ quality (i.e. steatosis, fibrosis, quality of perfusion), ischaemia times, donor and recipient characteristics, among others. Recent evidence from organ perfusion research of discarded organs has found the visual assessment of donated organs to be the most frequent reason for organ decline and discard in abdominal organ transplantation [11], [12], [13], [14], [15], [16].

The current method of visual assessment is subjective and largely reliant on surgeon experience. This leads to organ decline based on potentially inaccurate interpretations and inter-unit variability in organ acceptance [11], 14], 15], 17]. The emergence of easily accessible high quality cameras, in conjunction with novel artificial intelligence (AI) tools, has sparked interest in developing objective AI-powered organ quality assessment tools using retrieval photographs [9], 10], [18], [19], [20]. Previous studies have relied on manual image segmentation to isolate the organ from the background [7], 9], 18], 19]. This is time-consuming and prohibits development of automated and rapid quality assessment tools at the time of donation. Translation into clinical practice for any AI-based assessment tool will be significantly limited with image segmentation as the rate-limiting step.

We have addressed this issue by applying transfer learning techniques to develop automated deep learning image segmentation models for kidney and liver organ donation photographs. In addition, we compare the performance of these models with currently available software solutions for background removal. The study aims to evaluate organ segmentation performance between methods using intersection over union (IoU) as the primary outcome.

## Materials and methods

### Data

All images used for training and evaluating the segmentation models comprised photographs of organs ex situ after cold flush with preservation fluid, obtained at time of organ donation in the United Kingdom. They were all fully anonymised and used in accordance with the UK General Medical Council guidance for utilising images of internal organs or structures for secondary purposes [21]. Prior to their inclusion in the dataset, all images underwent manual pre-processing and selection. Selection criteria for inclusion encompassed a file size exceeding 100 kB, ensuring organ was within proper focus and clearly visible, maintaining appropriate exposure (neither excessively bright nor dark) and verifying an unobstructed view of the organ. The external validation sets for both kidneys and livers were distinct from their respective internal validation sets. They were collected by different UK retrieval (organ recovery) teams, using different cameras and spanning different years of retrieval activity.

Image annotation was performed using MakeSense.ai [22]. Each image received two annotations: ‘whole organ’ and ‘clear view’. The ‘whole organ’ annotation outlined the entire visible organ within the image. The ‘clear view’ annotation outlined only the parenchymal view of the organ, excluding vasculature, ducts and any other connective tissue. These were separated into two distinct single class annotations for subsequent steps. Annotations were converted into binary masks that served as the ground truth labels for subsequent assessment of segmentation performance.

### Segmentation methods and model development

A total of five segmentation methods were employed. Three were pre-developed background removal tools, including the Mac OS background removal tool (macBGRemoval) [23] and Python package rembg [24], utilising the isnet (remBGisnet) and u2net (remBGu2net) methods. It is important to note that since these methods primarily serve as background removal tools, they were exclusively employed for ‘whole organ’ segmentation and were not utilised for ‘clear view’ segmentation. We applied transfer learning with our custom train and internal validation datasets to develop two further novel deep learning image segmentation tools using open-source software (Detectron2 [25] and YoloV8 [26]). In these, we were able to train clear view models using our clear view annotations.

Detectron2 is an open-source object detection framework built by Facebook AI Research. It provides a flexible and modular platform for training and deploying computer vision models. One of the key features of Detectron2 is its support for pre-trained models, which accelerates development time and reduces the need for computational resources. For the training and internal validation of Detectron2, we utilised a pre-trained Mask R-CNN model with a ResNet-50-FPN backbone from Detectron2’s model zoo. The model weights were initialised using pre-trained weights provided by Detectron2. Our settings included processing images per batch of 2, with a base learning rate of 0.00025 and a batch size per image of 512. The determination of the maximum iterations for training was based on the analysis of training and validation loss curves. We selected an iteration where there was evidence of learning plateau and no indication of overfitting.

The YoloV8 algorithm represents a rapid, single-stage technique for object detection, comprising an input segment, backbone, neck and output segment. The input segment undertakes mosaic data augmentation, adaptive anchor calculation and adaptive grayscale padding on the input image. The backbone network and neck module serve as central components in the YoloV8 network, utilising them to extract feature maps at multiple scales from the input image. These feature maps are subsequently processed by the SPPF module, which employs pooling with varying kernel sizes to amalgamate the feature maps, prior to their transmission to the neck layer [27].

For YoloV8 training and testing, similarly to Detectron2, we utilised yolov8n-seg.pt, a pre-trained Yolo model specifically designed for segmentation tasks. The batch size was set to 4, and the image size was fixed at 800. Similar to the approach with Detectron2, we determined the maximum epochs for training based on the analysis of training and validation loss curves, selecting an iteration demonstrating a learning plateau and no signs of overfitting.

Segmented images from each of these methods were generated and converted into binary masks. These masks were then compared with the ground truth masks created at the end of the annotation process.

### Evaluation metrics

The primary outcome measure was Intersection over Union (IoU), which reflects the overlap between the predicted segmentation and the ground truth. Additionally, we measured secondary metrics including Dice Similarity Coefficient (DSC), Area Under the ROC Curve (AUROC), accuracy, precision and recall. These metrics were chosen in accordance with published guidelines for evaluating medical semantic image segmentation tasks [28], 29]. To assess the computational efficiency of our model, we measured the total time taken to perform segmentation on a directory of images. This total time was then divided by the number of images to calculate the average processing time per image.

### Hardware and software utilised

The experiments were conducted using a MacBook 16 Pro equipped with an M3 Pro chip and 18 GB of memory. The operating system was OS Sonoma 14.3.1. The software environment was Python 3.11.8 and PyTorch 2.2 for deep learning computations. We utilised Dectron2 v0.6, Yolo v8.1 and the isnet and u2net segmentation algorithms via remBG v2.0.56. Data analysis and visualisation were performed using R 4.3.3 with the tidyverse package.

We have made the YoloV8 and Detectron2 code used to develop these models available on GitHub to facilitate reproducibility and encourage its application in other medical photography contexts [30].

### Statistical analysis

Paired non-parametric tests were utilised to statistically compare the performance of our models. The Friedman test was used for tasks involving segmentation of entire organs, where there were more than two groups. Pairwise post hoc analysis using the Wilcoxon signed-rank test was used to identify differences between individual models. The Wilcoxon signed-rank test was also used for the clear view tasks where only two groups (Detectron2and YoloV8) were compared. Median and interquartile range results are shown unless specified. A p-value of <0.05 was considered statistically significant, except in post hoc testing where Bonferroni adjustment for multiple tests was applied and a p value of <0.0004 was considered statistically significant.

## Results

The kidney cohort included 1,024 images. Of these, 821 were used for training and internal validation, employing a 70/30 split (575 training images, 246 internal validation images). An additional 203 images from a separate photography collection were employed for external validation. The liver cohort comprised 608 images, with 400 images allocated for training and internal validation, employing a 70/30 split again (280 training images, 120 internal validation images). An additional 208 images from a separate photography collection were employed for external validation.

During model training, Detectron2 was trained for 3,000 iterations on both liver and kidney datasets, encompassing both whole organ and clear view images. Similarly, YoloV8 underwent training for 500 epochs for both whole kidney and whole liver segmentation tasks, with the validation loss plateauing and no signs of overfitting observed. Additionally, YoloV8 for clear kidney segmentation was trained for 235 epochs, while for clear liver, training continued up to 320 epochs, albeit with overfitting evident after epoch 95. Consequently, the model output from the 75th epoch was selected for further analysis due to its optimal performance (Supplementary Figures S3 and 6).

**Figure 1:**
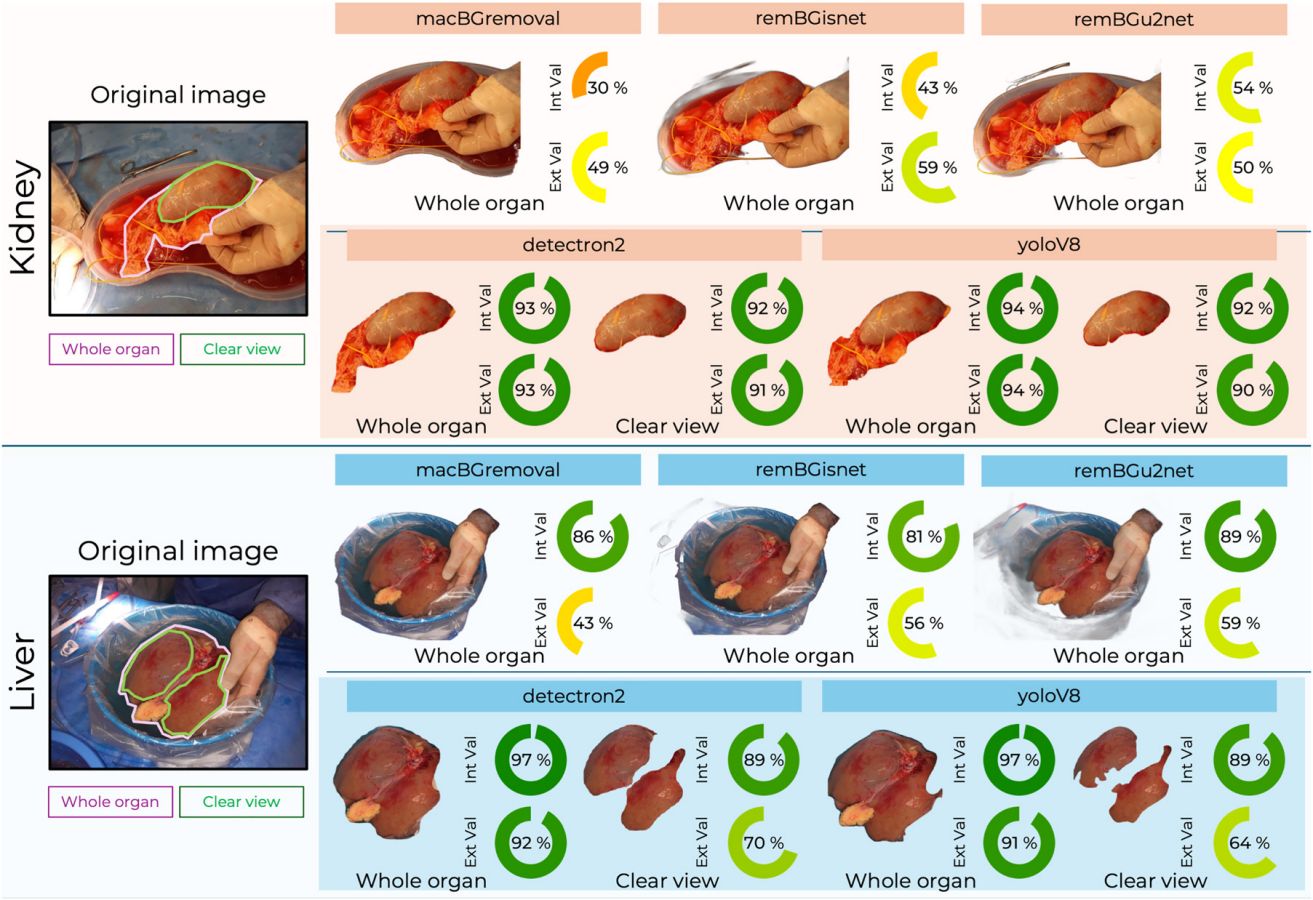
Median intersection over union (IoU) performance across segmentation methods for kidney and liver retrieval photographs in test and external validation datasets. Includes representative original images, without ground truth annotations, and segmentation outcomes for each method.

Examples of segmented images and associated median IoU performance scores for each tool are demonstrated in Figure 1.

### Kidney segmentation

**Figure 2:**
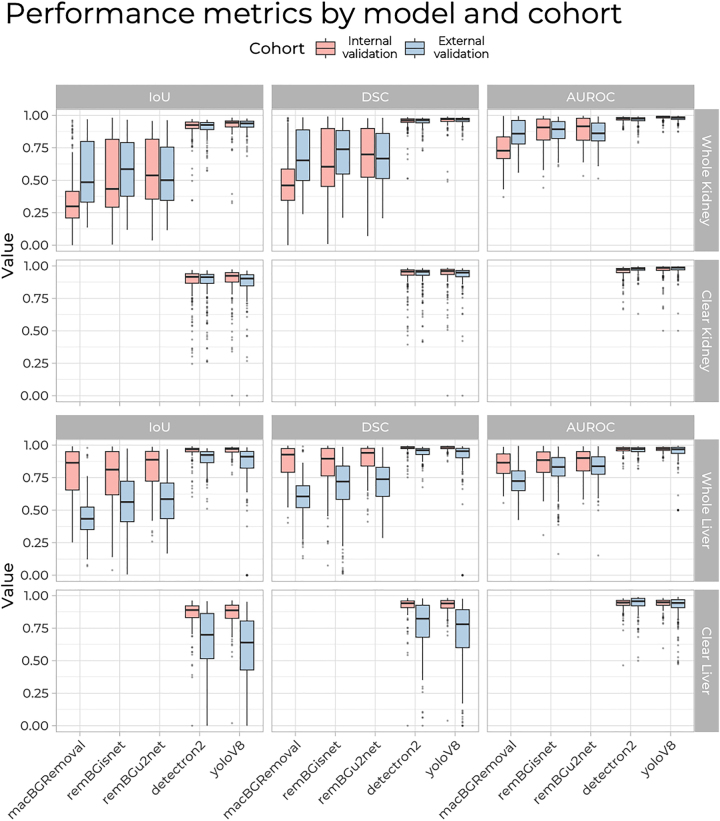
Comparative analysis of segmentation model performance with intersection over union (IoU), dice coefficient (DSC) and area under the receiver operating characteristic curve (AUROC). Box plots summarise the performance of the segmentation models across kidney and liver images.

In internal validation, both Detectron2 (IoU: 0.93) and YoloV8 (IoU: 0.94) achieved significantly better segmentation performance compared to macBGRemoval (IoU: 0.3), remBGisnet (IoU: 0.43), remBGu2net (IoU: 0.54), p<0.0001, Figure 2. Both Detectron2 and YoloV8 were found to significantly outperform the remaining tools (macBGRemoval, remBGisnet, remBGu2net) on post hoc analysis, p<0.0001 (Supplementary Figure S7). YoloV8 was the fastest method requiring only 0.31 s per image, compared with 1.10 s for Detectron2 (full results in Table 1). The other segmentation methods ranged from 0.69 s per image with macBGRemoval to 1.56 s per image with remBGisnet.

**Table 1:** Median (IQR) performance metrics and time to complete for all object detection models for kidney and liver segmentation.

Cohort	Segmentation	Metric	Model					*p*
			macBGRemoval	remBGisnet	remBGu2net	detectron2	yoloV8	
Internal validation	Whole kidney	IoU	0.3 (0.21–0.41)	0.43 (0.29–0.82)	0.54 (0.35–0.82)	0.93 (0.9–0.95)	0.94 (0.91–0.96)	<0.0001
n=246		DSC	0.46 (0.35–0.59)	0.6 (0.45–0.9)	0.7 (0.52–0.9)	0.96 (0.95–0.97)	0.97 (0.95–0.98)	<0.0001
		AUROC	0.73 (0.67–0.83)	0.91 (0.81–0.97)	0.92 (0.81–0.98)	0.98 (0.96–0.98)	0.99 (0.98–0.99)	<0.0001
		Time (sec)	169	383	314	271	76	–
		Time per image	0.69	1.56	1.28	1.10	0.31	–
	Clear view	IoU	–	–	–	0.92 (0.87–0.94)	0.92 (0.88–0.95)	0.001
		DSC	–	–	–	0.96 (0.93–0.97)	0.96 (0.93–0.97)	0.001
		AUROC	–	–	–	0.97 (0.95–0.98)	0.98 (0.97–0.99)	<0.001
		Time (sec)	–	–	–	179	66	–
		Time per image	–	–	–	0.73	0.27	–
External validation	Whole kidney	IoU	0.49 (0.33–0.8)	0.59 (0.38–0.79)	0.5 (0.35–0.76)	0.93 (0.89–0.94)	0.94 (0.91–0.96)	<0.0001
n=203		DSC	0.65 (0.5–0.89)	0.74 (0.55–0.88)	0.67 (0.51–0.86)	0.96 (0.94–0.97)	0.97 (0.95–0.98)	<0.0001
		AUROC	0.86 (0.78–0.96)	0.89 (0.82–0.95)	0.86 (0.8–0.94)	0.97 (0.96–0.98)	0.98 (0.97–0.99)	<0.0001
		Time (sec)	53	139	96	165	30	–
		Time per image	0.26	0.69	0.47	0.81	0.15	–
	Clear view	IoU	–	–	–	0.91 (0.87–0.93)	0.9 (0.85–0.93)	0.261
		DSC	–	–	–	0.95 (0.93–0.97)	0.95 (0.92–0.96)	0.271
		AUROC	–	–	–	0.98 (0.97–0.99)	0.99 (0.97–0.99)	<0.001
		Time (sec)	–	–	–	165	30	–
		Time per image	–	–	–	0.81	0.15	–
Internal validation	Whole liver	IoU	0.86 (0.66–0.95)	0.81 (0.62–0.95)	0.89 (0.72–0.95)	0.97 (0.95–0.98)	0.97 (0.95–0.98)	<0.0001
n=120		DSC	0.93 (0.79–0.97)	0.9 (0.76–0.97)	0.94 (0.84–0.98)	0.98 (0.97–0.99)	0.99 (0.97–0.99)	<0.0001
		AUROC	0.87 (0.78–0.93)	0.88 (0.79–0.95)	0.9 (0.8–0.95)	0.97 (0.96–0.98)	0.97 (0.96–0.98)	<0.0001
		Time (sec)	20	80	45	92	16	–
		Time per image	0.16	0.67	0.38	0.77	0.13	–
	Clear view	IoU	–	–	–	0.89 (0.83–0.92)	0.89 (0.83–0.93)	0.360
		DSC	–	–	–	0.94 (0.91–0.96)	0.94 (0.9–0.96)	0.327
		AUROC	–	–	–	0.95 (0.92–0.96)	0.95 (0.93–0.96)	0.920
		Time (sec)	–	–	–	91	15	–
		Time per image	–	–	–	0.76	0.13	–
External validation	Whole liver	IoU	0.43 (0.35–0.52)	0.56 (0.41–0.72)	0.59 (0.44–0.71)	0.92 (0.87–0.95)	0.91 (0.82–0.95)	<0.0001
n=208		DSC	0.61 (0.52–0.69)	0.72 (0.58–0.84)	0.74 (0.61–0.83)	0.96 (0.93–0.97)	0.95 (0.9–0.98)	<0.0001
		AUROC	0.72 (0.65–0.8)	0.83 (0.76–0.9)	0.84 (0.78–0.91)	0.97 (0.95–0.98)	0.97 (0.94–0.98)	<0.0001
		Time (sec)	178	368	330	248	79	–
		Time per image	0.86	1.77	1.59	1.19	0.38	–
	Clear view	IoU	–	–	–	0.7 (0.52–0.86)	0.64 (0.43–0.81)	<0.001
		DSC	–	–	–	0.82 (0.68–0.93)	0.78 (0.6–0.89)	<0.001
		AUROC	–	–	–	0.96 (0.92–0.98)	0.94 (0.91–0.97)	<0.001
		Time (sec)	–	–	–	310	70	–
		Time per image	–	–	–	1.49	0.34	–

IoU, intersection over union; DSC, dice coefficient; AUROC, area under the receiver operating characteristic curve. For comparisons between 2 groups, the Wilcoxon signed-rank test was used. For comparisons among more than 2 groups, the Friedman test was applied.

Similar results were observed for internal validation of clear view segmentation tasks. Both Detectron2 and YoloV8 models achieving an IoU of 0.92. YoloV8 was faster at 0.27 s per image compared to 0.72 s for Detectron2.

External validation showed similar results. There was no drop in performance for both Detectron2 (IoU: 0.93) and YoloV8 (IoU: 0.94), which again achieved performances significantly better than macBGRemoval (IoU: 0.49), remBGisnet (IoU: 0.59) and remBGu2net (IoU: 0.5), p<0.0001, see Figure 2. Both Detectron2 and YoloV8 again were found to significantly outperform the remaining tools on post hoc analysis, p<0.0001 (Supplementary Figure S7). YoloV8 remained the fastest method, with a processing time of 0.15 s per image, while Detectron2 required 0.81 s per image. The other methods required from 0.26 s per image with macBGRemoval to 0.69 s per image with remBGisnet (full results in Table 1).

**Figure 3:**
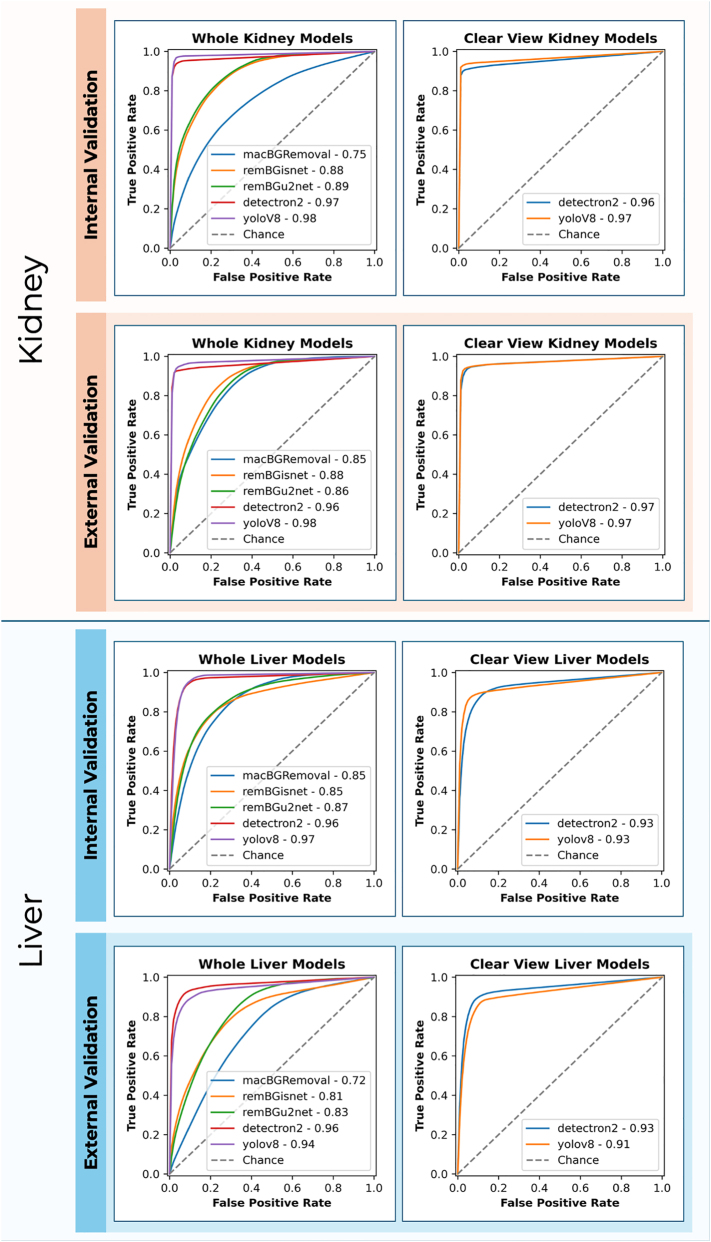
Receiver operating characteristic (ROC) curves comparing the performance of the image segmentation methods for object detection in retrieval photographs. Performance metrics are indicated for both internal and external validation phases, with ‘chance’ representing a baseline random classifier.

External validation of clear view segmentation tasks saw both Detectron2 and YoloV8 yield an IoU of 0.91 and 0.90 respectively, p=0.261. In terms of processing time, YoloV8 maintained a shorter processing time at 0.15 s per image, compared to Detectron2 at 0.81 s per image. The AUROC values for Detectron2 and YoloV8 were also high, at 0.98 and 0.99, respectively, Figure 3.

### Liver segmentation

In internal validation, Detectron2 and YoloV8 showed superior performance in whole liver segmentation with an IoU of 0.97 for both. They significantly outperformed macBGRemoval (IoU: 0.86), remBGisnet (IoU: 0.81) and remBGu2net (IoU: 0.89), p<0.0001, Figure 2. Both Detectron2 and YoloV8 were found to significantly outperform the remaining tools on post hoc analysis, p<0.0001 (Supplementary Figure S7). YoloV8 was the most time-efficient taking only 0.13 s per image, compared to Detectron2, which required 0.77 s per image. The other methods ranged from 0.16 s per image for macBGRemoval and 0.67 s per image for remBGisnet (full results in Table 1).

Similar results were observed in the internal validation of clear view segmentation tasks for the liver. Detectron2 and YoloV8 both achieving an IoU of 0.89, p=0.360. YoloV8 maintained a shorter processing time at 0.13 s per image, compared to Detectron2 at 0.76 s per image. No significant differences were observed in the AUROC, with both methods showing a value of 0.95, p=0.920, Figure 3.

External validation for whole liver segmentation confirmed the strong performance of Detectron2 (IoU: 0.92) and YoloV8 (IoU: 0.91). Both significantly outperformed macBGRemoval (IoU: 0.43), remBGisnet (IoU: 0.56) and remBGu2net (IoU: 0.59), p<0.0001, as indicated in Figure 2. Both Detectron2 and YoloV8 again significantly outperformed the remaining tools on *post hoc* analysis, p<0.0001 (Supplementary Figure S7). YoloV8 remained the fastest method, requiring 0.38 s per image, while Detectron2’s processing time was 1.19 s per image. The other methods took longer, with macBGRemoval requiring 0.86 s per image and remBGisnet taking 1.77 s per image (complete results are given in Table 1).

In external validation of clear view liver segmentation, Detectron2 and YoloV8 showed a difference in performance. Detectron2 achieved an IoU of 0.7 and YoloV8 an IoU of 0.64, p<0.001. We manually checked images with IoU of <0.5 to investigate any obvious reasons for this drop in performance. The livers in these pictures were all submerged in preservation fluid, with a small surface area of the liver clearly visible, compared to the remaining pictures in this cohort. Example images can be seen in Supplementary Figure S8. In terms of processing time, YoloV8 was faster at 0.34 s per image compared to 1.49 s for Detectron2.

## Discussion

This research presents the first novel deep learning–based approach for achieving fully automated, high-accuracy semantic segmentation of kidneys and livers in medical photographs captured during organ donation. Our method precisely delineates organ boundaries and extracts clear views of the parenchyma, all within 1.5 s per image. This is the first time such a method has been developed and externally validated on two separate datasets of images from within the United Kingdom.

Ultimately, this technology will enable efforts in developing automated and objective organ quality assessment tools to assist decision making. Macroscopic assessment of steatosis for livers and quality of perfusion for kidneys by surgical teams is routine and the most common documented reason for organ discard [11], [12], [13], [14]. Recent evidence for organ perfusion research found that 35 % of livers were declined due to a visual assessment of steatosis [13], while 38–43 % of kidneys were declined due to a visual assessment of inadequate perfusion [15], 16]. These visual assessments remain poorly defined with inconsistent inter-rater variability [11], 14], 17] leading to inappropriate organ discard [31].

While histological assessment is still considered the gold standard, its limitations include limited availability, time constraints, sampling bias and variability between observers [32], [33], [34], [35]. The Banff Working Group on Liver Allograft Pathology recently published a study on the inconsistencies of current steatosis assessment of frozen sections of donor livers. It highlighted a lack of standardised criteria for the assessment of donor liver biopsies from fresh frozen section [32]. There remains an unmet clinical need for an objective and efficient point-of-care decision support tool.

Previous literature on image segmentation for isolating organs in medical photographs has relied on manual techniques [7], 9], 18], 19]. Our group previously used a colour-based pixel analysis approach for identifying the organ parenchyma. This approach was very specific, with a high false negative rate. In the presence of blood-stained preservation fluid around the organ, this approach would sometimes identify the water as the organ (Supplementary Figure S9).

One previous publication [20] reported using machine learning methods for image segmentation, achieving an accuracy of 89 % with precision and recall of 97 %. Both Detectron2 and YoloV8 models achieved superior performance, exceeding accuracies of 96 %, precisions of 97 % and recall of 97 % (Supplementary Tables S1 and S2). We note that we did not employ these metrics as our primary outcomes. This is because previous research has shown that these metrics can overestimate segmentation performance, particularly recall. Consequently, they are not ideal for differentiating the performance of various segmentation methods. Recent guidance highlights Intersection over Union (IoU) and Dice Similarity Coefficient (DSC) as the key metrics for evaluating semantic segmentation tasks [28], 29], as such they were used in our study.

The task of clear view segmentation is entirely novel and represents a significant advancement in the field. Unlike traditional segmentation methods, clear view segmentation goes beyond simply outlining the entire organ. It can extract pixels that correspond to a clear view of the organ’s parenchyma. This refined segmentation steps holds potential for computer vision tasks in organ transplantation. By isolating the parenchyma, subsequent image analysis can concentrate on this critical area, leading to more precise assessments and potentially improved transplant outcomes.

A notable benefit of incorporating image segmentation models into deep learning classifiers is in explainable AI. It has been observed that image classifiers sometimes base their decisions on background elements rather than the foreground, leading to potentially misleading outcomes. In unpublished preliminary research conducted by our team, we explored classifiers designed to determine whether an organ was suitable for transplantation. We discovered that the algorithms performed exceptionally well by identifying the type of gloves in the image – sterile theatre gloves indicated a transplanted organ, whereas non-sterile blue gloves signified organs not accepted and photographed in a research setting. However, when the background was eliminated, the performance of the algorithms significantly decreased. Introducing an image segmentation step not only maintains human oversight in subsequent processing but also provides a clear explanation of which parts of the image the AI analysed. This transparency is crucial in validating and trusting AI-generated findings.

We acknowledge that comparing our deep learning models to background removal software is not a perfect equivalence. From a practical standpoint, however, there are currently no other automated options for isolating organs from photographs at time of organ recovery, or retrieval. This has been demonstrated by the reliance of prior research in having to resort to manual segmentation techniques. Given the absence of a more directly comparable automated method, we employed background removal software as the nearest available alternative to benchmark our organ segmentation models against.

An additional limitation is that we were unable to capture individual processing times for each image. This restricted us to comparisons between groups using only average processing time data. While average processing times provide a general benchmark for segmentation speed, they can mask variations that might exist between individual images. Having access to individual processing times would allow for a more nuanced analysis of factors that might influence segmentation speed. For instance, it would be informative to explore how image file size impacts processing times. It is encouraging to note that all deep learning methods achieved segmentation times under 1.5 s on average, indicating a very fast processing speed. This rapid processing suggests that segmentation time is unlikely to be a major bottleneck in real-world computer vision applications. This is a critical factor in the field of organ transplantation where time for decision making is constrained by the necessity to minimise organ ischaemic times.

Future research efforts will expand the applicability of these deep learning tools. One example is applying similar methodologies to segment *in situ* liver photographs. Livers look different before and after cold preservation flushing, making this particularly important in the assessment of liver steatosis, the most common cause for organ decline. This could significantly enhance the utility of this technique within the transplantation workflow, building upon previous attempts at *in situ* liver analysis [20] and leading to an increase in organ utilisation. Another area of work for enhancing the future applicability of these models involves the verification and validation processes required for certification. This would enable their approved use in clinical settings. Currently, these algorithms are employed solely for research purposes and have not yet been certified for clinical application.

This segmentation methodology may also be applied in the segmentation of biliary and vascular structures. In kidney transplantation, a picture of the aortic patch is commonly shared to visualise the extent of atherosclerosis and the kidney’s vascular anatomy, including any aberrant polar artery anatomy. In liver transplantation, vascular and biliary anatomy can vary greatly between donors, making these factors crucial in planning the implanting operation. Accurate visualisation and segmentation can enable surgeons to anticipate and prepare for the transplant recipient operation.

Additional applications to other organs, like the pancreas, are also possible. Segmenting the pancreas may be more challenging due to its parenchymal similarity to surrounding tissues, but overcoming this will greatly benefit image analysis algorithm development for pancreas photographs. Finally, this methodology can be further developed to enable video analysis and segmentation either in real-time or on existing video files.

## Supplementary Material

Supplementary Material
