# Supplementary material for: Deep learning for automated boundary detection and segmentation in organ donation photography
Source: Innov Surg Sci. 2024 Aug 20;10(3):131–41. doi: 10.1515/iss-2024-0022 (PMC7617812; doi:10.1515/iss-2024-0022)
Supplement: Supplementary file 1 — Supplementary Material [file j_iss-2024-0022_suppl_001.pdf]

Deep learning for automated boundary detection and segmentation in organ donation photography

Georgios Kourounis, Ali Ahmed Elmahmudi, Brian Thomson, Robin Nandi, Samuel J. Tingle, Emily K. Glover, Emily Thompson, Balaji Mahendran, Chloe Connelly, Beth Gibson, Lucy Bates, Neil S. Sheerin, James Hunter, Hassan Ugail and Colin Wilson

Supplementary material

Supplementary Table S1. Summary of all median (IQR) results between internal and external validation kidney cohorts.

Supplementary Table S2. Summary of all median (IQR) results between internal and external validation liver cohorts.

Supplementary Figure S1: Kidney segmentation model performance.

Supplementary Figure S2: Summary Receiver Operating Characteristic Curves for all segmentation models in both kidney whole organ and clear view image segmentation tasks.

Supplementary Figure S3: Line graphs showing training and external validation segmentation loss from each model for kidney whole organ and clear view segmentation tasks.

Supplementary Figure S4: Liver segmentation model performance.

Supplementary Figure S5: Summary Receiver Operating Characteristic Curves for all segmentation models in both liver whole organ and clear view image segmentation tasks.

Supplementary Figure S6: Line graphs showing training and external validation segmentation loss from each model for liver whole organ and clear view segmentation tasks.

Supplementary Figure S7: Comparative analysis of segmentation model performance for whole organ segmentation with pairwise post-hoc test analyses.

Supplementary Figure S8: Example of liver photographs where segmentation most challenging.

Supplementary Figure S9: Colour-based pixel analysis for distinguishing liver parenchyma in retrieval photographs.

Supplementary Table S1. Summary of all median (IQR) performance results between internal and external validation kidney cohorts.

Cohort	Segmentation	Metric	Model					*p*
			macBGRemoval	remBGisnet	remBGu2net	detectron2	yoloV8	
Internal validation<br><br>n= 246	Whole Kidney	IoU	0.3 (0.21-0.41)	0.43 (0.29-0.82)	0.54 (0.35-0.82)	0.93 (0.9-0.95)	0.94 (0.91-0.96)	<0.0001
		DSC	0.46 (0.35-0.59)	0.6 (0.45-0.9)	0.7 (0.52-0.9)	0.96 (0.95-0.97)	0.97 (0.95-0.98)	<0.0001
		AUROC	0.73 (0.67-0.83)	0.91 (0.81-0.97)	0.92 (0.81-0.98)	0.98 (0.96-0.98)	0.99 (0.98-0.99)	<0.0001
		Accuracy	0.74 (0.58-0.85)	0.85 (0.71-0.97)	0.88 (0.71-0.97)	0.99 (0.98-0.99)	0.99 (0.99-0.99)	<0.0001
		Precision	0.33 (0.22-0.5)	0.45 (0.29-0.85)	0.55 (0.35-0.84)	0.97 (0.96-0.98)	0.96 (0.94-0.97)	<0.0001
		Recall	0.96 (0.62-1)	1 (0.99-1)	1 (1-1)	0.96 (0.94-0.97)	0.99 (0.97-0.99)	<0.0001
		Time (seconds)	169	383	314	271	76	-
		Seconds per image	0.69	1.56	1.28	1.10	0.31	-
	Clear View	IoU	-	-	-	0.92 (0.87-0.94)	0.92 (0.88-0.95)	0.001
		DSC	-	-	-	0.96 (0.93-0.97)	0.96 (0.93-0.97)	0.001
		AUROC	-	-	-	0.97 (0.95-0.98)	0.98 (0.97-0.99)	<0.001
		Accuracy	-	-	-	0.99 (0.99-1)	1 (0.99-1)	0.001
		Precision	-	-	-	0.98 (0.96-0.99)	0.96 (0.93-0.97)	<0.001
		Recall	-	-	-	0.94 (0.9-0.96)	0.97 (0.94-0.98)	<0.001
		Time (seconds)	-	-	-	179	66	-
		Seconds per image	-	-	-	0.73	0.27	-

External Validation<br>n= 203	Whole Kidney	IoU	0.49<br>(0.33-0.8)	0.59<br>(0.38-0.79)	0.5<br>(0.35-0.76)	0.93<br>(0.89-0.94)	0.94<br>(0.91-0.96)	<0.0001
		DSC	0.65<br>(0.5-0.89)	0.74<br>(0.55-0.88)	0.67<br>(0.51-0.86)	0.96<br>(0.94-0.97)	0.97<br>(0.95-0.98)	<0.0001
		AUROC	0.86<br>(0.78-0.96)	0.89<br>(0.82-0.95)	0.86<br>(0.8-0.94)	0.97<br>(0.96-0.98)	0.98<br>(0.97-0.99)	<0.0001
		Accuracy	0.78<br>(0.66-0.96)	0.84<br>(0.74-0.94)	0.8<br>(0.69-0.93)	0.98<br>(0.98-0.99)	0.99<br>(0.98-0.99)	<0.0001
		Precision	0.49<br>(0.33-0.83)	0.63<br>(0.39-0.83)	0.5<br>(0.35-0.8)	0.98<br>(0.96-0.98)	0.97<br>(0.95-0.98)	<0.0001
		Recall	1<br>(1-1)	1<br>(0.99-1)	1<br>(1-1)	0.96<br>(0.93-0.97)	0.98<br>(0.96-0.99)	<0.0001
		Time (seconds)	53	139	96	177	32	-
		Seconds per image	0.26	0.69	0.47	0.87	0.16	-
	Clear View	IoU	-	-	-	0.91<br>(0.87-0.93)	0.9<br>(0.85-0.93)	0.261
		DSC	-	-	-	0.95<br>(0.93-0.97)	0.95<br>(0.92-0.96)	0.271
		AUROC	-	-	-	0.98<br>(0.97-0.99)	0.99<br>(0.97-0.99)	<0.001
		Accuracy	-	-	-	0.99<br>(0.99-1)	0.99<br>(0.99-1)	0.504
		Precision	-	-	-	0.95<br>(0.91-0.97)	0.93<br>(0.9-0.96)	0.010
		Recall	-	-	-	0.97<br>(0.95-0.98)	0.98<br>(0.95-0.99)	<0.001
		Time (seconds)	-	-	-	165	30	-
		Seconds per image	-	-	-	0.81	0.15	-

IoU - Intersection over Union, **DSC** - Dice Coefficient, **AUROC** - Area Under the Receiver Operating Characteristic curve.
For comparisons between 2 groups, the Wilcoxon signed-rank test was used. For comparisons among more than 2 groups, the Friedman test was applied.

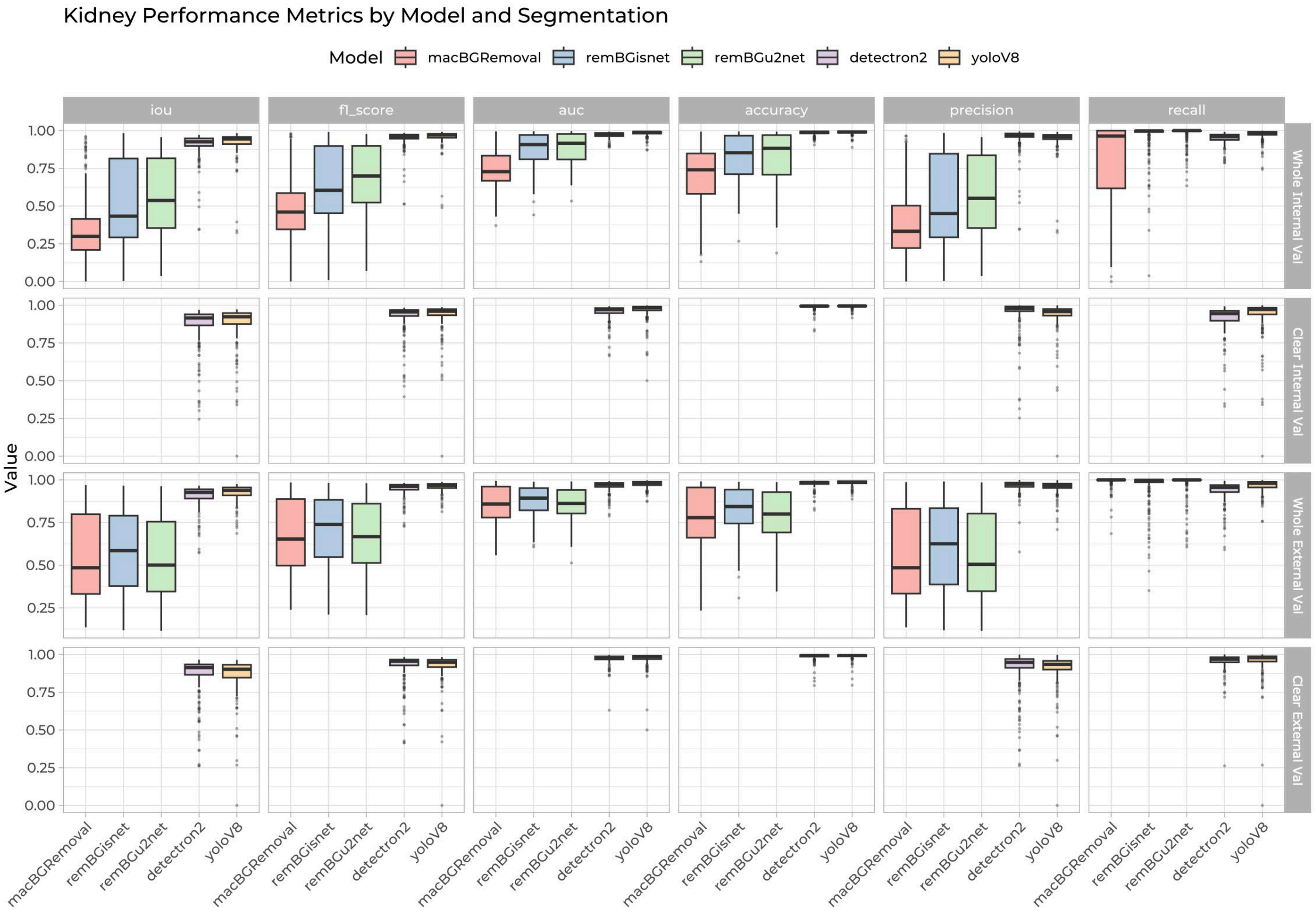

Supplementary Figure S1: Kidney segmentation model performance. Box plots display performance metrics (IoU, F1 score, AUC, accuracy, precision, recall) for kidney segmentation models across internal and external validation cohorts. Each metric is analysed within Whole Kidney and Clear Kidney categories.

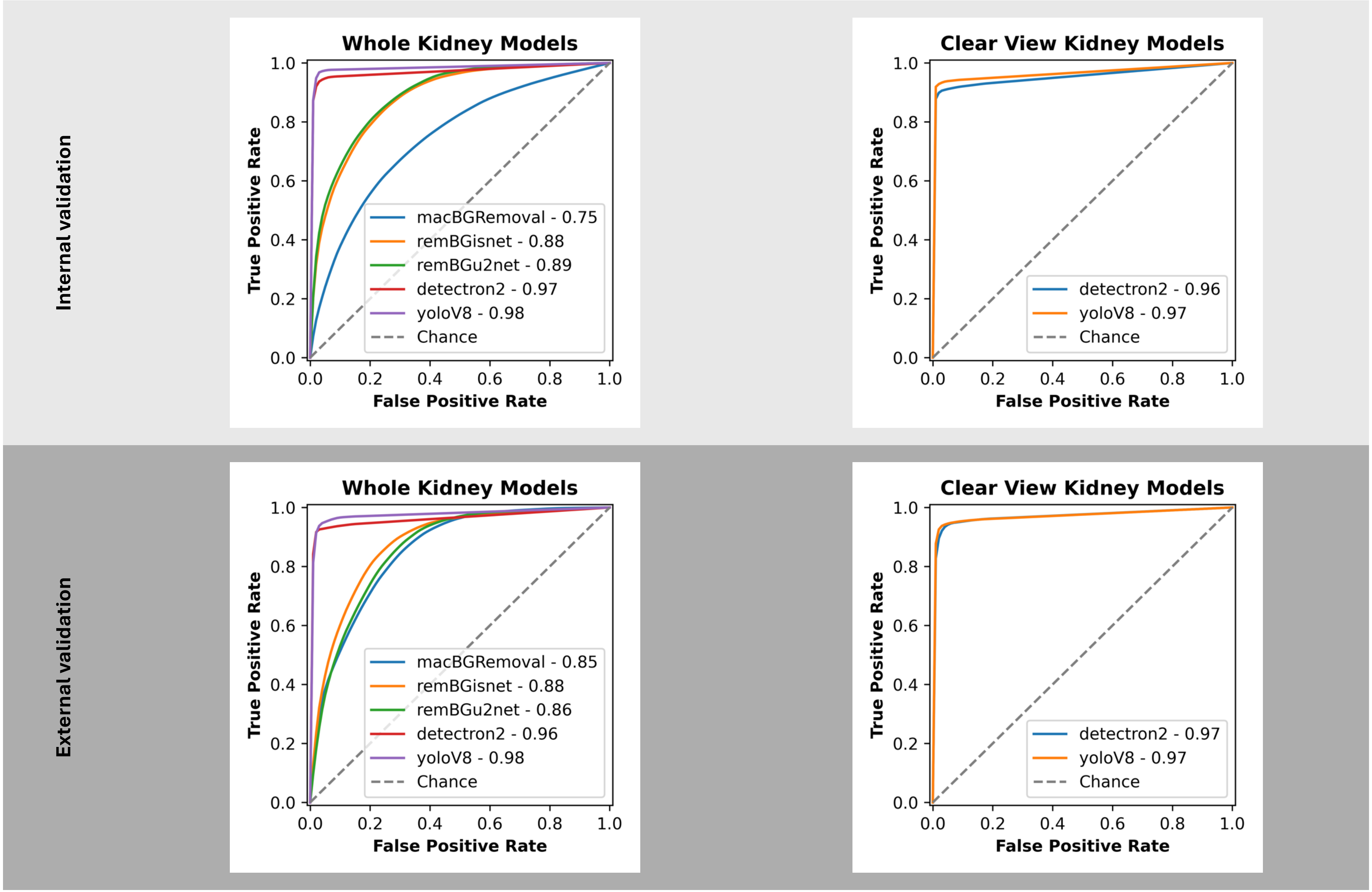

Supplementary Figure S2: Summary ROC Curves for all segmentation models in both kidney whole organ and clear view image segmentation tasks.

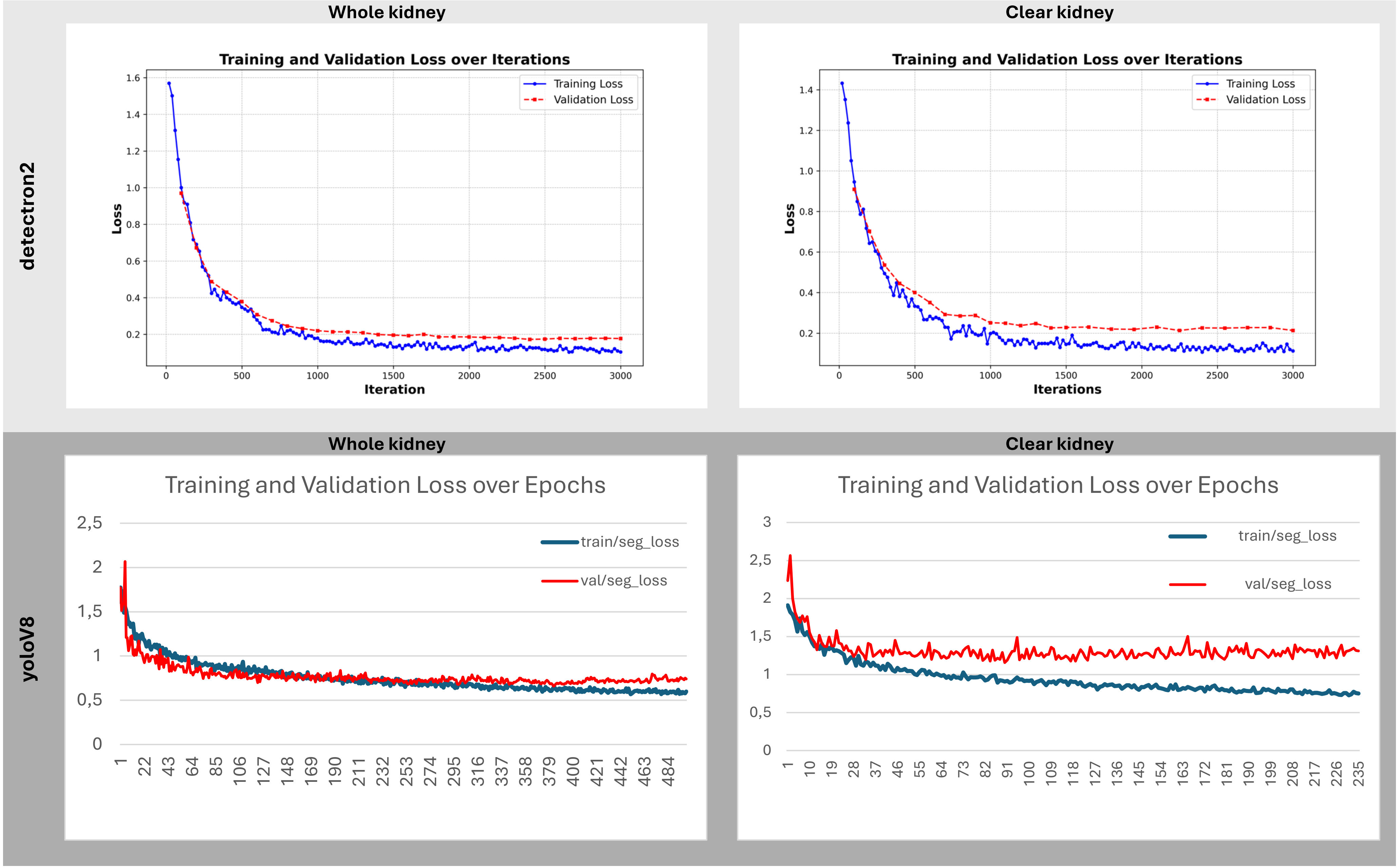

Supplementary Figure S3: Line graphs showing training and internal validation segmentation loss from each model for kidney whole organ and clear view segmentation tasks.

Supplementary Table S2. Summary of all median (IQR) results between internal and external validation liver cohorts.

Cohort	Segmentation	Metric	Model					p
			macBGRemoval	remBGisnet	remBGu2net	detectron2	yoloV8	
Internal validation<br>n= 120	Whole Liver	IoU	0.86 (0.66-0.95)	0.81 (0.62-0.95)	0.89 (0.72-0.95)	0.97 (0.95-0.98)	0.97 (0.95-0.98)	<0.0001
		DSC	0.93 (0.79-0.97)	0.9 (0.76-0.97)	0.94 (0.84-0.98)	0.98 (0.97-0.99)	0.99 (0.97-0.99)	<0.0001
		AUROC	0.87 (0.78-0.93)	0.88 (0.79-0.95)	0.9 (0.8-0.95)	0.97 (0.96-0.98)	0.97 (0.96-0.98)	<0.0001
		Accuracy	0.91 (0.75-0.96)	0.89 (0.76-0.96)	0.91 (0.81-0.97)	0.98 (0.97-0.98)	0.98 (0.97-0.99)	<0.0001
		Precision	0.88 (0.71-0.97)	0.95 (0.81-0.98)	0.94 (0.8-0.97)	0.98 (0.97-0.99)	0.98 (0.97-0.98)	<0.0001
		Recall	1 (1-1)	0.99 (0.81-1)	1 (0.96-1)	0.99 (0.97-0.99)	1 (0.99-1)	<0.0001
		Time (seconds)	20	80	45	92	16	-
		Seconds per image	0.16	0.67	0.38	0.77	0.13	-
	Clear View	IoU	-	-	-	0.89 (0.83-0.92)	0.89 (0.83-0.93)	0.360
		DSC	-	-	-	0.94 (0.91-0.96)	0.94 (0.9-0.96)	0.327
		AUROC	-	-	-	0.95 (0.92-0.96)	0.95 (0.93-0.96)	0.920
		Accuracy	-	-	-	0.95 (0.93-0.97)	0.96 (0.94-0.97)	0.119
		Precision	-	-	-	0.95 (0.9-0.98)	0.98 (0.95-0.99)	<0.001
		Recall	-	-	-	0.94 (0.92-0.96)	0.92 (0.88-0.95)	<0.001
		Time (seconds)				91	15	-
		Seconds per image				0.76	0.13	-
External Validation	Whole Liver	IoU	0.43 (0.35-0.52)	0.56 (0.41-0.72)	0.59 (0.44-0.71)	0.92 (0.87-0.95)	0.91 (0.82-0.95)	<0.0001

n= 208		DSC	0.61 (0.52-0.69)	0.72 (0.58-0.84)	0.74 (0.61-0.83)	0.96 (0.93-0.97)	0.95 (0.9-0.98)	<0.0001
		AUROC	0.72 (0.65-0.8)	0.83 (0.76-0.9)	0.84 (0.78-0.91)	0.97 (0.95-0.98)	0.97 (0.94-0.98)	<0.0001
		Accuracy	0.64 (0.57-0.73)	0.78 (0.7-0.88)	0.78 (0.69-0.88)	0.98 (0.96-0.99)	0.97 (0.94-0.99)	<0.0001
		Precision	0.46 (0.36-0.55)	0.61 (0.46-0.84)	0.59 (0.44-0.71)	0.98 (0.94-0.99)	0.96 (0.9-0.98)	<0.0001
		Recall	1 (0.89-1)	0.99 (0.85-1)	1 (0.99-1)	0.96 (0.94-0.98)	0.97 (0.94-0.99)	<0.0001
		Time (seconds)	178	368	330	248	79	-
		Seconds per image	0.86	1.77	1.59	1.19	0.38	-
	Clear View	IoU	-	-	-	0.7 (0.52-0.86)	0.64 (0.43-0.81)	<0.001
		DSC	-	-	-	0.82 (0.68-0.93)	0.78 (0.6-0.89)	<0.001
		AUROC	-	-	-	0.96 (0.92-0.98)	0.94 (0.91-0.97)	<0.001
		Accuracy	-	-	-	0.96 (0.93-0.98)	0.95 (0.92-0.97)	<0.001
		Precision	-	-	-	0.73 (0.55-0.89)	0.67 (0.47-0.86)	<0.001
		Recall	-	-	-	0.97 (0.9-0.99)	0.95 (0.9-0.98)	0.030
		Time (seconds)				310	70	-
		Seconds per image				1.49	0.34	-

IoU - Intersection over Union, **DSC** - Dice Coefficient, **AUROC** - Area Under the Receiver Operating Characteristic curve.
For comparisons between 2 groups, the Wilcoxon signed-rank test was used. For comparisons among more than 2 groups, the Friedman test was applied.

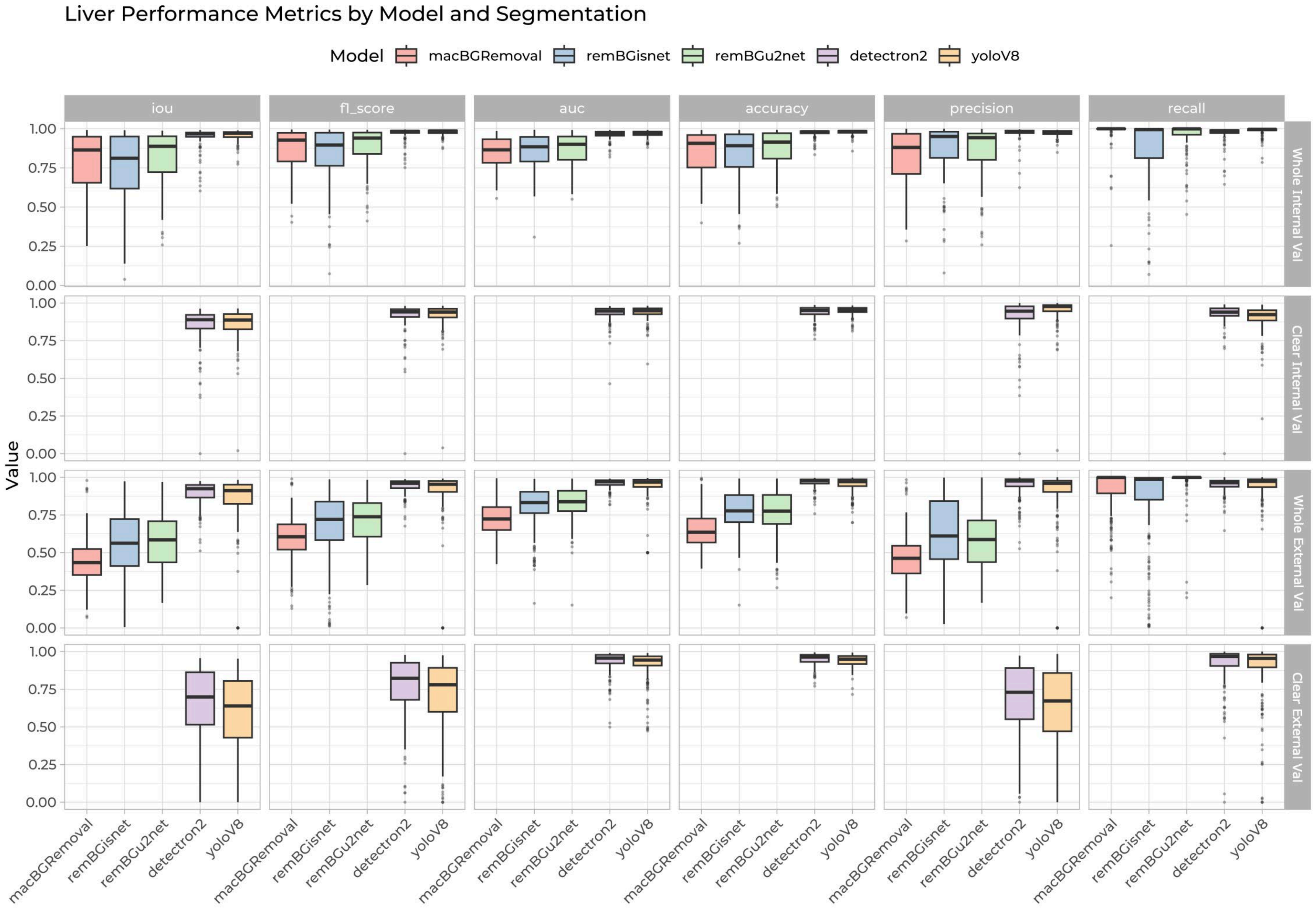

Supplementary Figure S4: Liver segmentation model performance. Box plots display performance metrics (IoU, F1 score, AUC, accuracy, precision, recall) for kidney segmentation models across internal and external validation cohorts. Each metric is analysed wit hin Whole Kidney and Clear Kidney categories.

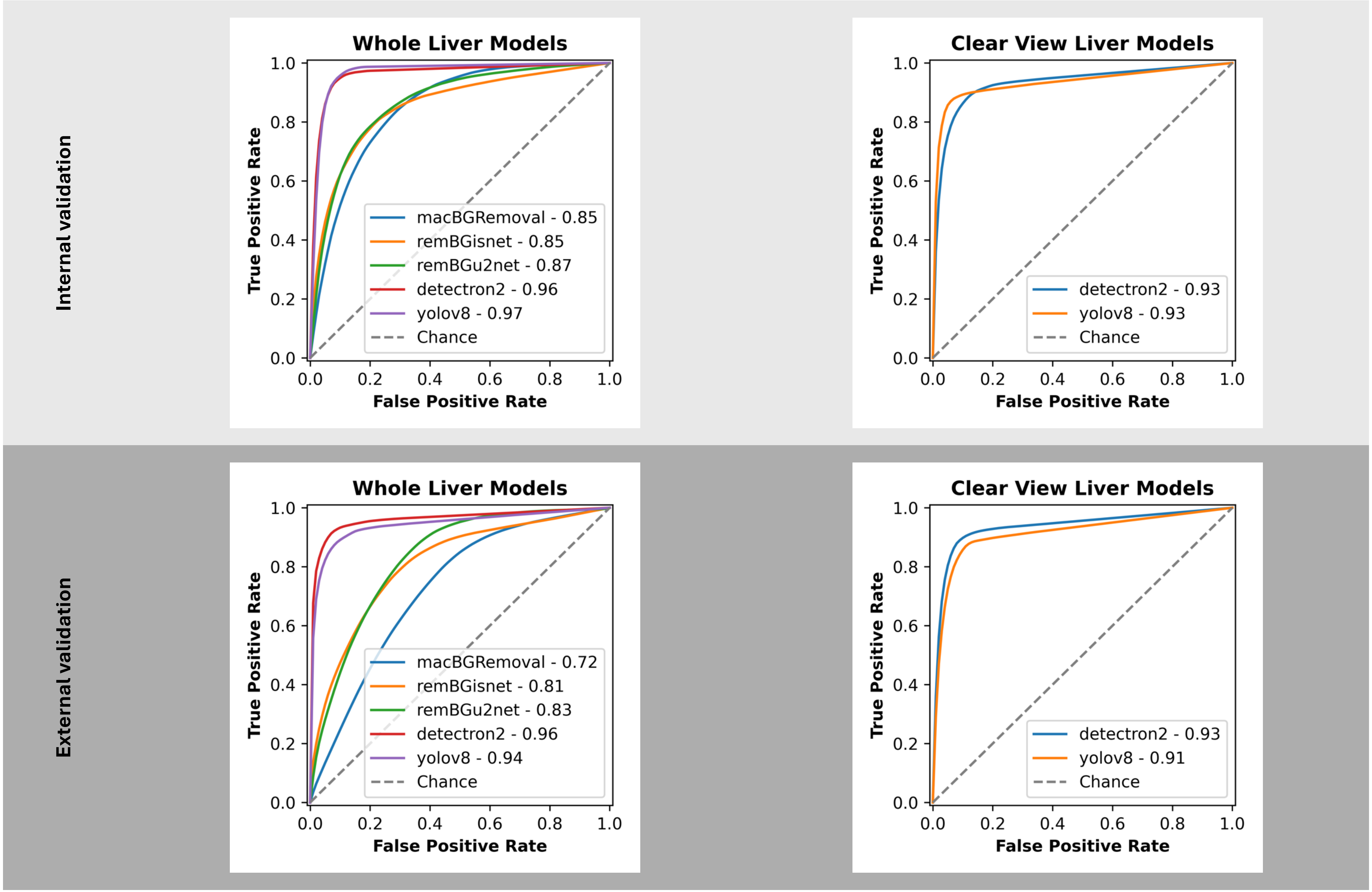

Supplementary Figure S5: Summary ROC Curves for all segmentation models in both liver whole organ and clear view image segmentation tasks.

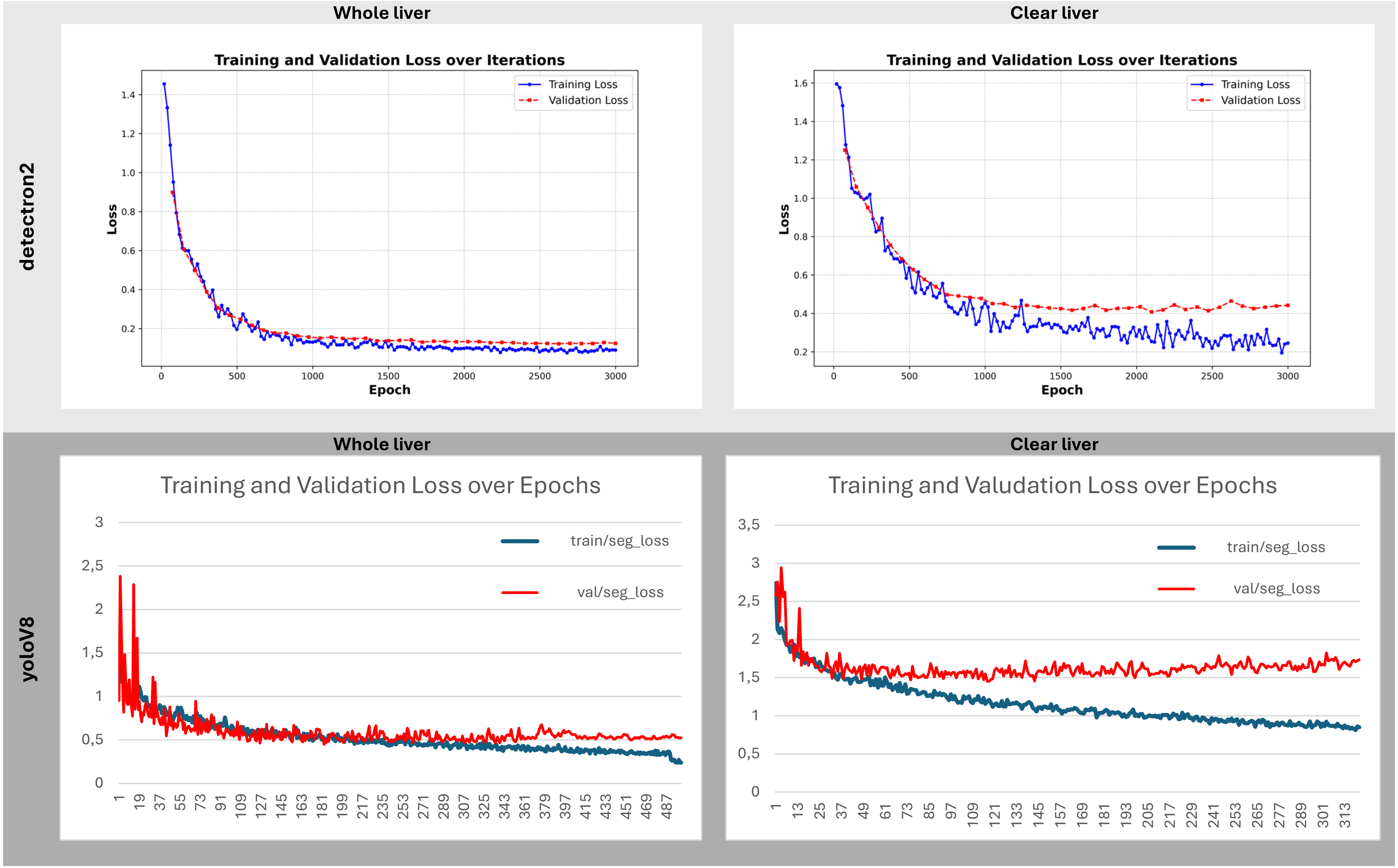

Supplementary Figure S6: Line graphs showing training and internal validation segmentation loss from each model for liver whole organ and clear view segmentation tasks

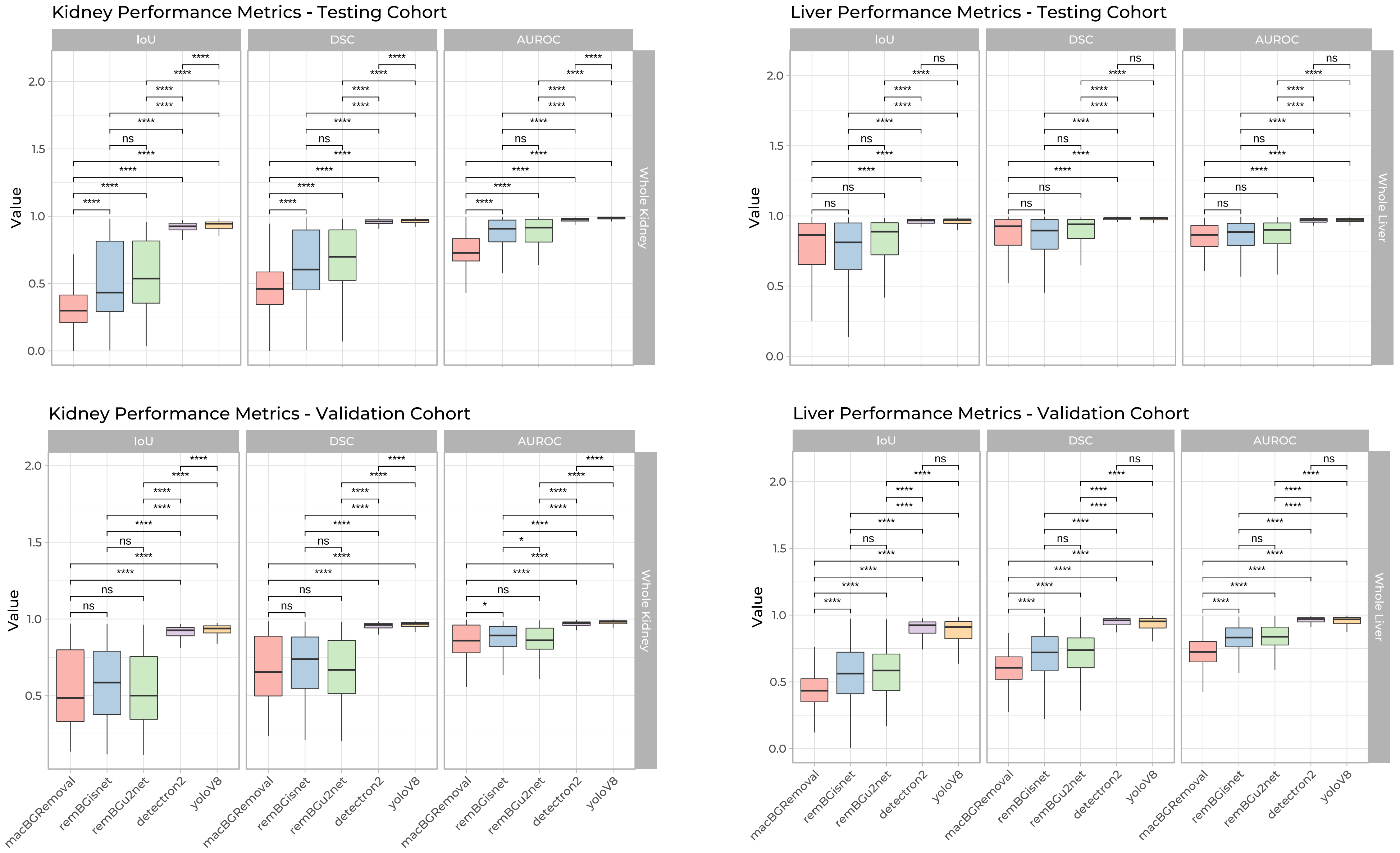

Supplementary Figure S7: Comparative analysis of segmentation model performance for whole organ segmentation with pairwise post-hoc test analyses. Intersection over Union (IoU), Dice Coefficient (DSC), and Area Under the Receiver Operating Characteristic curve (AUROC). Box plots summarise the performance of the segmentation models across kidney and liver images. * $p < 0.05$, ** $p < 0.01$, *** $p < 0.001$, **** $p < 0.0001$.

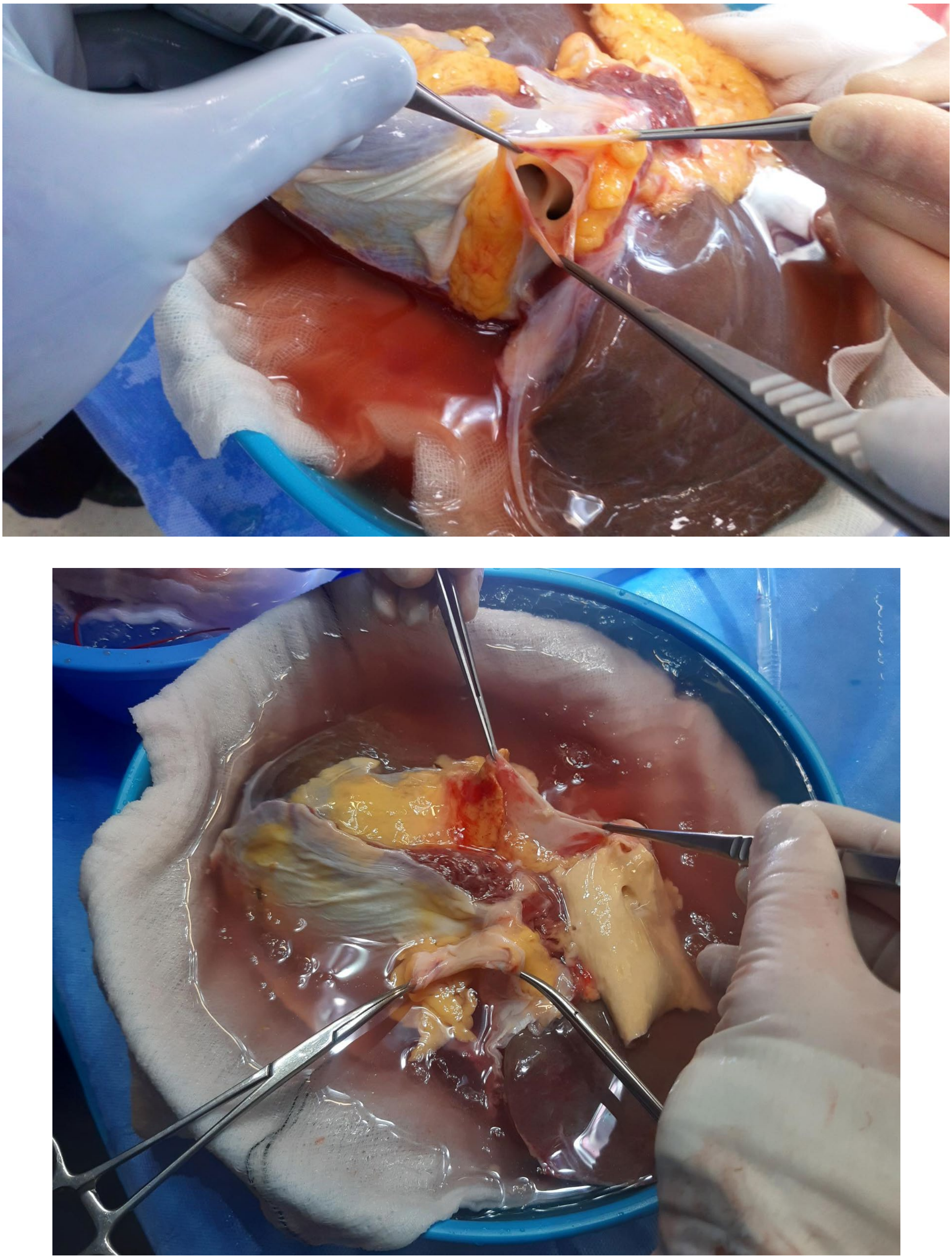

Supplementary Figure S8: Example of liver photographs where segmentation most challenging, with Clear Liver segmentation IoU results of <0.5.

A

B

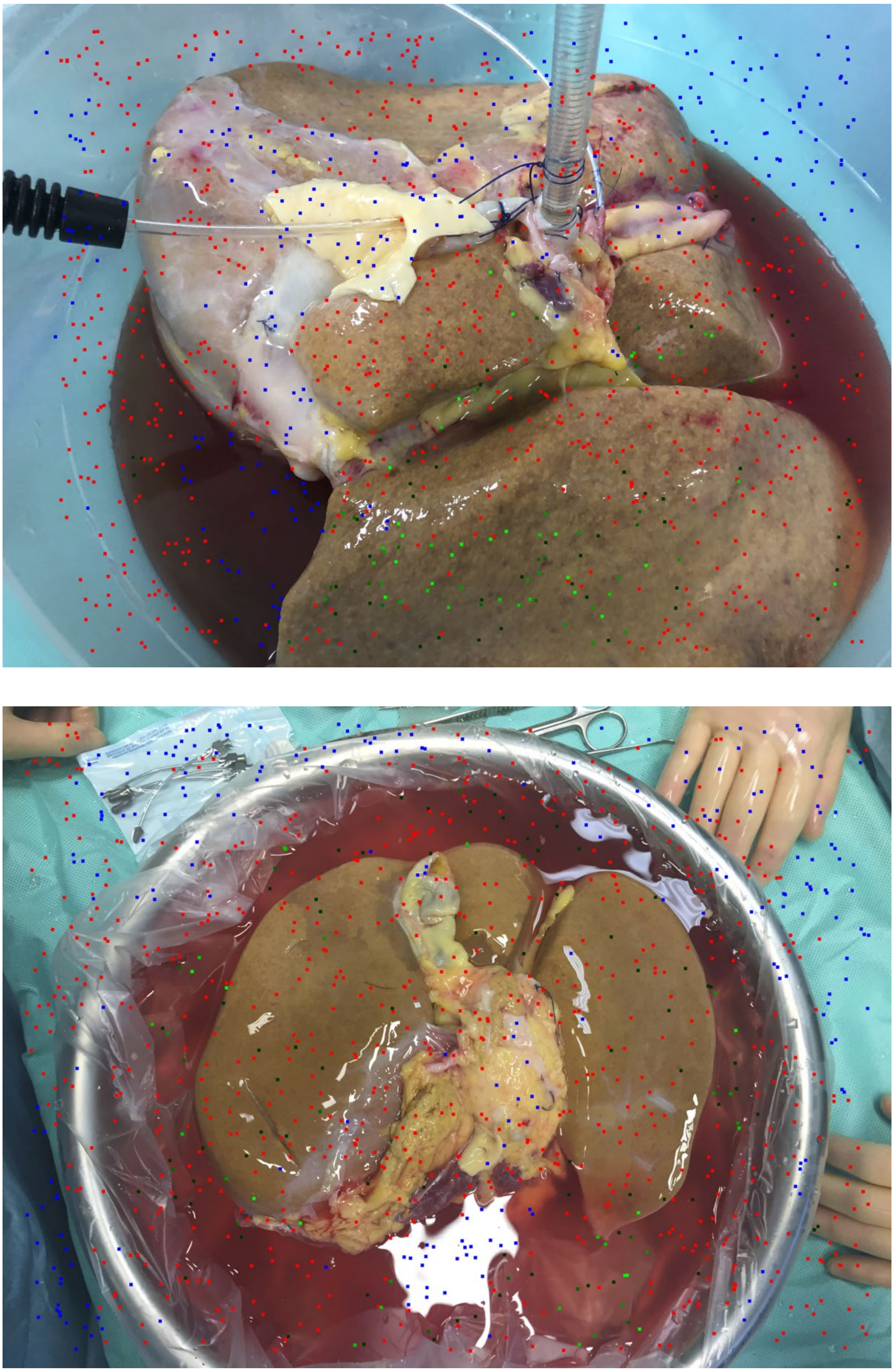

Supplementary Figure S9: Colour-based pixel analysis for distinguishing liver parenchyma in retrieval photographs. Green dots indicate areas classified as liver parenchyma, while red and blue dots denote non-parenchymal regions. (A) Liver where all green dots correctly identifying liver tissue, but majority of dots on liver erroneously identified as not liver (B) Liver with all green dots are on blood-stained preservation fluid, with all liver areas marked in red or blue.
